# Clozapine-induced myoclonus in a patient with treatment-resistant schizophrenia: A case report

**DOI:** 10.1192/j.eurpsy.2026.11776

**Published:** 2026-07-31

**Authors:** E. Maaloul, I. Chaari, M. Kessentini, W. Abid, F. Charfeddine, L. Aribi, N. Messedi, J. Aloulou

**Affiliations:** Psychiatric department “B”, Hedi Chaker University Hospital, Sfax, Tunisia

## Abstract

**Introduction:**

Clozapine is widely used for treatment-resistant schizophrenia but can cause neurological adverse effects, including myoclonus, that may challenge long-term treatment adherence and effectiveness.

**Objectives:**

To describe clozapine-associated myoclonus and examine the relationship between dose, plasma level, and symptom onset.

**Methods:**

Single-patient case report conducted in the “B” psychiatric department, Hedi Chaker University Hospital, Sfax, Tunisia. The patient was hospitalized in July 2025. Clinical observations, routine laboratories, dosing history, and a clozapine plasma level at symptom onset were extracted from the medical record. Data was de-identified.

**Results:**

We present the case of a 44-year-old woman with treatment-resistant schizophrenia was involuntarily admitted for behavioral disturbances. This was her second psychiatric hospitalization. The symptomatology was dominated by a persecutory delusion directed toward her family. After non-response to multiple antipsychotics, clozapine was initiated and titrated over two months to 300 mg/day with standard monitoring. Shortly after reaching this dose, involuntary myoclonic jerks appeared. At onset, the plasma clozapine level was 349 ng/mL, near the lower therapeutic range. The clozapine dose was reduced to 250 mg/day and clonazepam 1 mg was added, leading to complete resolution of myoclonus.

**Conclusions:**

Clozapine can precipitate myoclonus even at plasma levels within or near the lower therapeutic range.

Plasma monitoring and treatment adjustment should be considered when neurological adverse effects emerge to balance efficacy and tolerability.

**Disclosure of Interest:**

None Declared

